# The link between chronic kidney disease and cardiovascular disease

**DOI:** 10.12860/jnp.2014.19

**Published:** 2014-07-01

**Authors:** Sarmad Said, German T. Hernandez

**Affiliations:** Division of Nephrology & Hypertension, Department of Internal Medicine, Paul L. Foster School of Medicine, Texas Tech University Health Sciences Center at El Paso, El Paso, Texas, USA

**Keywords:** Cardiovascular disease, Chronic kidney disease, Hypertension, Diabetes mellitus

## Abstract

*Context:* It is well known that patients with chronic kidney disease (CKD) have a strong risk of cardiovascular disease (CVD). However, the excess risk of cardiovascular disease in patients with CKD is only partially explained by the presence of traditional risk factors, such as hypertension and diabetes mellitus.

*Evidence Acquisitions:* Directory of Open Access Journals (DOAJ), Google Scholar, PubMed, EBSCO and Web of Science has been searched.

*Results:* Chronic kidney disease even in its early stages can cause hypertension and potentiate the risk for cardiovascular disease. However, the practice of intensive blood pressure lowering was criticized in recent systematic reviews. Available evidence is inconclusive but does not prove that a blood pressure target of less than 130/80 mmHg as recommended in the guidelines improves clinical outcomes more than a target of less than 140/90 mmHg in adults with CKD.

*Conclusions:* The association between CKD and CVD has been extensively documented in the literature. Both CKD and CVD share common traditional risk factors, such as smoking, obesity, hypertension, diabetes mellitus, and dyslipidemia. However, cardiovascular disease remains often underdiagnosed und undertreated in patients with CKD. It is imperative that as clinicians, we recognize that patients with CKD are a group at high risk for developing CVD and cardiovascular events. Additional studies devoted to further understand the risk factors for CVD in patients with CKD are necessary to develop and institute preventative and treatment strategies to reduce the high morbidity and mortality in patients with CKD.

## 1. Context

It is well known that patients with chronic kidney disease (CKD) have a strong risk of cardiovascular disease (CVD) (1). However, the excess risk of cardiovascular disease in patients with CKD is only partially explained by the presence of traditional risk factors, such as hypertension and diabetes mellitus. We must look beyond traditional CVD risk factors to be able to develop and institute risk-lowering interventions to improve the health of our patients with CKD.

## 2. Evidence Acquisitions

Directory of Open Access Journals (DOAJ), Google Scholar, PubMed, EBSCO and Web of Science has been searched.

## 3. Results

The association between CKD and CVD was first reported by Dr. Bright in 1836 (2). Impairment in renal function can increase the risk of CVD two- to fourfold (3). CKD is considered present when impaired kidney function is confirmed in two or more occasions at least 3 months apart (4). The estimated glomerular filtration rate (eGFR) can be calculated using serum creatinine and the chronic kidney disease epidemiology collaboration (CKD-EPI) equation (5). Assessment for proteinuria is determined by the urinary albumin-to-creatinine ratio (5). Based on these measurements CKD is categorized in 5 levels of GFR and three stages of proteinuria (4).

Various studies have demonstrated that low eGFR and increased albuminuria are associated with a higher incidence of CVD. The cardiovascular mortality in patients with stage 3 CKD was twofold higher and threefold higher in patients with stage 4 CKD when compared to patients with normal renal function (6,7). The risk of developing congestive heart failure (CHF), atrial fibrillation, stroke, coronary heart disease (CAD), and peripheral artery disease (PAD) is increased two fold in patients with eGFR<60 mL/min/1.73m^2^(8-12). Recent meta-analyses have demonstrated that impaired renal function could be considered as an independent risk factor for development of CVD (13,14).

Two large cohort studies reported markedly decreased life expectancies for patients with CKD stage 3B (a 17-year shorter survival) and CKD stage4 (a 25-year shorter survival) compared with subjects with normal kidney function (15). Patients with CKD and CVD have a higher mortality rate(58-71%) compared with patients with CVD and normal renal function (22-27.5%) (16). The impact of the of CKD on CVD risk appears to be stronger when compared to traditional cardiovascular risk factors such as diabetes mellitus and hypertension as the reported reduction in life expectancy for middle-aged patients with diabetes mellitus and hypertension is approximately 8 and 3 years respectively (17-20).

Hypertension is a strong risk factor for the development of CKD. Kokubo *et al*., have shown that hypertension increases the incidence of CVD in subjects with CKD more than in patients with normal kidney function (21). The prevalence of left ventricular hypertrophy (LVH) in patients with CKD is increased; especially in patients with an eGFR<30 mL/min/1.73 m^2^ in whom the risk of developing new LVH is increased by 50%, which may partially explain the increased prevalence of sudden cardiac death in this population (59 deaths/1000 CKD person-years vs. 1 death/100 person-years in patients without CKD) (22,23).

CKD even in its early stages can cause hypertension and potentiate the risk for CVD. However, the practice of intensive blood pressure lowering was criticized in recent systematic reviews. Available evidence is inconclusive but does not prove that a blood pressure target of less than 130/80 mmHg as recommended in the guidelines improves clinical outcomes more than a target of less than 140/90 mmHg in adults with CKD (19).

In 2012, Canadian hypertension education program guidelines made a significant change to its target BP for patients with non-diabetic chronic kidney disease, increasing the target from <130/80 mmHg to <140/90 mmHg, whereas those with kidney disease and concomitant diabetes continue to have a target of <130/80 mmHg (20).

Data have shown that statins are effective anti-inflammatory drugs that lower cardiovascular event rates. Since inflammation is highly prevalent in patients with CKD and the risk of cardiovascular events increases dramatically with declining eGFR, statins should be especially beneficial in this patient group (24).

Valvular heart disease and atherosclerosis are more prevalent in patients with end-stage kidney disease and also in earlier stages of CKD (25). An imbalance between inhibitors of vascular calcification (such as fetuin-A and matrix Gla protein) and stimulators of calcification (hyperphosphatemia, elevated serum calcium-phosphate product) as well as leptin may play an essential role in increasing the risk of valvular disease and atherosclerosis (25).

Other factors associated with CKD can increase the risk for CVD. The renin-angiotensin and the sympathetic nervous systems are over stimulated and result in the increased production of superoxide, interleukin 6, and other pro-inflammatory cytokines (26). Also the activity of renalase, an enzyme produced by the kidneys that inactivates catecholamines, is decreased in patients with CKD (26).

The association between CKD and acute coronary syndrome (ACS) has been demonstrated in multiple studies (27-29). Among patients with ACS who also have CKD, the mortality is increased twofold compared to patients with ACS and normal kidney function (30). The adverse effect of CKD on the mortality of patients with ACS should be considered as a strong motivator to develop of new strategies from well-organized research to reduce the burden of risk in this population and accomplish improved outcomes. Large registry studies have shown that 40% of patients with non-ST-elevation myocardial infarction (NSTEMI), and 30% of subjects with ST-elevation myocardial infarction (STEMI), have an underlying CKD, as defined by an estimated glomerular filtration rate (eGFR) <60 mL/min per 1.73 m^2^ (31,32). However, a significant decrease in utilization of coronary angiography among patients with CKD has been reported. In a study of 85,743 elderly patients with acute MI, patients who had CKD underwent coronary angiography at nearly 50% less compared with subjects with normal or near-normal renal function. A significant reduction in the odds of death was observed among patients with CKD and MI who had an indication for angiography and actually had coronary angiography performed (33).

Diabetes mellitus is the most common cause of CKD worldwide (34). The progression of diabetic CKD is strongly associated with the duration of diabetes (35). Current recommendations suggest an overall glycemic control goal to a hemoglobin A1c (HbA1c) level of <7.0% to prevent diabetic CKD and to reduce the risk of CVD (36,37). However, for diabetic patients with established CKD stages 3 to 4, there is new data suggesting that HbA1c levels below 6.5% are associated with an increased risk of death. Therefore, it seems reasonable that, for diabetic patients with established CKD, glycemic control goals should target HbA1c level of no less than 7% (38).

The identification of clinical manifestations of CVD is challenging in the presence of CKD. The atypical presentations of ACS in patients with CKD should raise the clinical awareness to avoid under diagnosing potentially life-threatening cardiovascular events. This consideration is relevant because CKD patients (particularly for CKD stages 3-5) have higher rates of comorbidities, conduction abnormalities, and anterior infarctions compared with individuals without CKD (39). Cardiac troponins (I and T) are more sensitive than creatine phosphokinase-MB for detection of acute myocardial infarction in the general population, however the sensitivity might be reduced in CKD patients. Cardiac troponin concentrations are frequently increased in patients with CKD, which limits their use as biomarkers for ACS (40). The reluctance to perform coronary angiography in patients with CKD, especially CKD stages 3-5, has led to an underdiagnosis of atherosclerotic disease in this population (41). Subsequently, percutaneous coronary intervention and coronary artery bypass grafting tend to be underused in patients with CKD (42). Often,the presence of CKD leads to underutilization of angiography, PCI, and CABG and it may also extend to underutilization of secondary prevention strategies. Fox *et al.* have shown that even the prescription of statins, β blockers, and antiplatelet agents is less frequent for patients with acute myocardial infarct and CKD even though the treatment benefits have been recommended and by the 2012 European guidelines on cardiovascular disease prevention (43,44). Nevertheless, further systematic research is required to expand our understanding of the pathophysiology of non-traditional CVD risk factors in patients with CKD to eventually develop appropriate prevention and therapeutic approaches.

To prevent CVD events in patients with CKD, treatment should be initiated during the early stages of CKD. The presence of albuminuria is a predictive factor for the progression of CKD and is associated with an increased risk of CVD, even in the setting of normal renal function (45). Among patients with type 2 diabetes and microalbuminuria multimodal intervention including strict glucose management, statins, antihypertensive agents, aspirin, and lifestyle modification compared with standard therapies showed a reduction in vascular complication as well as cardiovascular and all-cause mortality (46, 47).

## 4. Conclusions

The association between CKD and CVD has been extensively documented in the literature. Both CKD and CVD share common traditional risk factors, such as smoking, obesity, hypertension, diabetes mellitus, and dyslipidemia. However, cardiovascular disease remains often underdiagnosed und undertreated in patients with CKD. It is imperative that as clinicians, we recognize that patients with CKD are a group at high risk for developing CVD and cardiovascular events. Additional studies devoted to further understand the risk factors for CVD in patients with CKD are necessary to develop and institute preventative and treatment strategies to reduce the high morbidity and mortality in patients with CKD.

## Authors’ contributions

All authors wrote the manuscript equally.

## Conflict of interests

The author declared no competing interests.

## Funding/Support

This research received no specific grant from any funding agency in the public, commercial, or not-for-profit sectors
